# Ellipsoid calculations versus manual tumor delineations for glioblastoma tumor volume evaluation

**DOI:** 10.1038/s41598-022-13739-4

**Published:** 2022-06-22

**Authors:** Clara Le Fèvre, Roger Sun, Hélène Cebula, Alicia Thiery, Delphine Antoni, Roland Schott, François Proust, Jean-Marc Constans, Georges Noël

**Affiliations:** 1 Department of Radiotherapy, ICANS, Institut Cancérologie Strasbourg Europe, 17 Rue Albert Calmette, 67200 Strasbourg Cedex, France; 2 Department of Radiotherapy, Institut Gustave Roussy, Paris-Saclay University, Villejuif, France; 3 Department of Neurosurgery, Hôpital d’Hautepierre, 1, Avenue Molière, 67200 Strasbourg, France; 4 Department of Public Health, ICANS, Institut Cancérologie Strasbourg Europe, 17 Rue Albert Calmette, 67200 Strasbourg Cedex, France; 5 Department of Medical Oncology, ICANS, Institut Cancérologie Strasbourg Europe, 17 Rue Albert Calmette, 67200 Strasbourg Cedex, France; 6 Department of Radiology, Centre Hospitalier Universitaire d’ Amiens, 1 Rond-Point du Professeur Christian Cabrol, 80054 Amiens Cedex 1, France

**Keywords:** Cancer imaging, Cancer, Neurology, Oncology

## Abstract

In glioblastoma, the response to treatment assessment is essentially based on the 2D tumor size evolution but remains disputable. Volumetric approaches were evaluated for a more accurate estimation of tumor size. This study included 57 patients and compared two volume measurement methods to determine the size of different glioblastoma regions of interest: the contrast-enhancing area, the necrotic area, the gross target volume and the volume of the edema area. The two methods, the ellipsoid formula (the calculated method) and the manual delineation (the measured method) showed a high correlation to determine glioblastoma volume and a high agreement to classify patients assessment response to treatment according to RANO criteria. This study revealed that calculated and measured methods could be used in clinical practice to estimate glioblastoma volume size and to evaluate tumor size evolution.

## Introduction

Glioblastoma (GBM) is the most common and aggressive brain tumor in adults. Although the prognosis of GBM patients improved with the introduction of adjuvant chemoradiotherapy protocol, it remains poor, with a median overall survival (OS) of 15–18 months^1^. The conventional gadolinium-enhanced magnetic resonance imaging (MRI) is the gold standard radiological examination to the assessment to treatment with the tumor size monitoring. An optimal tumor size evaluation is primordial to evaluate progression and propose the most relevant therapeutic strategy.

The evaluation of the response to treatment is essentially based on tumor size evolution, which is often used as an endpoint of clinical studies^2^. Traditionally, tumor size is estimated by a cross-sectional 2D method with the product of the largest perpendicular diameters on T1-weighted contrast-enhanced MRI rather than a 1D method^3^. In 1990, MacDonald et al*.* were the first to propose criteria for treatment response assessment with the enhancing tumor area 2D measurements evolution^4^. In 2009, the RANO working group published the RANO criteria and defined four groups of GBM treatment response: complete response, partial response, stable disease, and progressive disease. The RANO radiological criteria included the tumor size evolution in 2D obtained by calculating the sum of the product of the largest diameters on measurable lesions (at least 10 mm) and ranked a tumor as “progression disease” when the 2D size increased at least 25% or when the fluid-attenuation inversion recovery on T2-weighted (T2-FLAIR) images lesion increased but was without measurement guidelines^5^.

This size measurement method was criticized because GBM is usually an irregular tumor with a cystic area, a surgical cavity, hemorrhage and no sharp demarcation that could compromise the size estimation and lead to error in therapeutic decisions^6–8^. Consequently, the volumetric approach seemed to be more appropriate to obtain a better estimation of tumor size with more accuracy^6^. In 2017, Ellingson et al*.* published modified RANO criteria to estimate GBM evolution. In addition to the two-dimensional measurement, a volumetric approach was described and an increase of 40% or more of the total tumor volume on two sequential MRI separated with 4–8 weeks defined a “durable progression disease”^9^. However, no detail of the measurement method was described^9,10^.

With improvements in imaging techniques, higher resolution, complex tumors such as GBM are easily and precisely measured in size. However, according to the method of measurement, the cost, the expertise, the complexity or the time required to reach results are highly variable^7,11^. Many possibilities are available to measure the volume of a tumor: manual segmentation, semi-automated segmentation, automated segmentation or calculation methods^12^. Despite the numerous and heterogeneous tumor volume estimation advanced techniques currently available, their use in daily medical practice remains limited due to lack of resources and time. Nowadays, no segmentation algorithm had demonstrated its superiority to the others in term of volume measurement accuracy. The accessibility to all practitioners is restricted and often only radiologists used it. For that, to discuss patient management, simple but less precise 1D or 2D methods are often employed.

To improve accuracy and reproducibility of measurements, volumetric approach is necessary. The ellipsoid model was proposed as an acceptable alternative simple volumetric measurement method to replace the 1D and 2D methods. The ellipsoid method uses the three orthogonal linear diameters of the tumor. Some authors compared different geometric model as spheroid, ellipsoid, cylinder or rectangular models and concluded that ellipsoid model was the best for the tumor volume appreciation^13,14^. Other complex methods of tumor volume measurements in glioblastoma were studied as manual segmentation, semiautomated segmentation or fully automated segmentation with discordant results^15–19^. Although numerous publications on semi or automated segmentation models exist in the recent literature, algorithms are heterogeneous and lack of standardization and availability^15,20–22^. Complementary researches and uniformity are required. Even if manual segmentation is a time-consuming method which can lead to bias and inter-observer variability^15,22^, authors showed the participation of a neurooncologist/radiologist expert for the manual segmentation allowed a higher accuracy than automated segmentation for tumor size determination^23^.

This study compared two methods of volume measurement, the ellipsoid model and the manual segmentation, to estimate the size of different GBM regions of interest in adult patients with the aim to propose an acceptable method of volume measurement, available for all, reproductible, simple and easy to use in clinical routine situations.

## Results

### Calculated volume (CV) versus measured volume (MV)

The analysis of the calculated and measured volume is summarized in Table 1.

**Table 1:** Calculated volume and measured volume for the different glioblastoma compartments (114 MRIs).

	Calculated volume (CV) (diameters 3D) (cm^3^)	Measured volume (MV) (delineation) (cm^3^)	Difference CV − MV (cm^3^)	((CV − MV)/CV)*100 (%)		CV (diameters 3D) (cm^3^)	MV (delineation) (cm^3^)	Difference CV − MV (cm^3^)	((CV − MV)/CV)*100 (%)
**CE total**					**CE before CRT**				
Mean Median Range	41.6 27.9 0.6 to 167.6	15.5 11.2 0.3 to 66.0	26.1 17.9 −0.2 to 117.3	174.9 162.1 −26.1 to 398.5	Mean Median Range	37.1 26.7 2.4 to 167.6	12.6 9.2 1.2 to 66.0	24.9 18.7 0.8 to 117.3	196.6 189.3 6.9 to 398.5
					**CE at suspicion of progression**				
					Mean Median Range	46.1 31.0 0.6 to 159.6	18.3 11.5 0.3 to 60.7	27.8 17.5 −0.2 to 115.3	153.2 145.3 −26.10 to 392.1
**NEC total**					**NEC before CRT**				
Mean Median Range	17.5 6.3 0.0 to 128.1	7.3 2.4 0.0 to 42.4	10.1 3.0 −0.1 to 95.9	150.3 111.3 −10.7 to 789.3	Mean Median Range	15.4 3.6 0.0 to 128.1	5.9 1.5 0.0 to 39.8	9.6 1.7 −0.1 to 95.9	165.4 125.6 −10.7 to 789.3
					**NEC at suspicion of progression**				
					Mean Median Range	19.5 9.9 0.0 to 106.6	8.8 3.6 0.0 to 42.4	10.7 5.3 0.0 to 70.3	139.9 100.8 20.8 to 615.8
**GTV total**					**GTV before CRT**				
Mean Median Range	58.7 52.7 1.2 to 145.8	47.6 39.8 1.0 to 128.2	11.1 8.8 −28.9 to 67.0	26.1 22.1 −68.8 to 207.2	Mean Median Range	54.7 47.0 5.1 to 145.0	42.8 36.4 4.1 to 107.9	11.9 9.0 −12.7 to 53.6	30.5 26.4 −24.1 to 117.6
					**GTV at suspicion of progression**				
					Mean Median Range	62.2 55.6 1.2 to 145.8	52.4 53.2 1.0 to 128.2	10.3 6.00 −28.9 to 67.0	21.7 15.0 −68.8 to 207.2
**FLAIR total**					**FLAIR before CRT**				
Mean Median Range	179.2 142.0 13.7 to 722.9	132.3 110.8 13.6 to 520.2	46.9 32.4 −53.3 to 222.5	38.2 34.2 −34.5 to 172.2	Mean Median Range	129.7 94.5 13.7 to 505.3	95.9 73.4 13.6 to 339.3	33.8 23.9 −50.4 to 189.1	38.5 32.1 −34.5 to 172.1
					**FLAIR at suspicion of progression**				
					Mean Median Range	228.7 200.1 30.7 to 722.9	168.8 146.3 21.3 to 520.2	59.9 50.6 −53.3 to 222.5	37.9 39.0 −29.5 to 113.3

Calculated volume (CV) and measured volume (MV) and difference between the two measurement methods for the CE, the NEC, the GTV, and the FLAIR compartments for the 114 MRIs: the 57 MRIs before CRT and the 57 MRIs showing a suspicion of progression.

*CE* contrast-enhancement, *GTV* gross target volume, *NEC* necrosis area.

CV was significantly larger than MV for each tumor compartment, the contrast-enhancing area (CE), the necrotic area (NEC), the gross target volume (GTV) and the volume of the edema area (FLAIR) (CE p < 0.001, NEC p = 0.01, GTV p = 0.05 and FLAIR p = 0.01).

**Figure 1:**
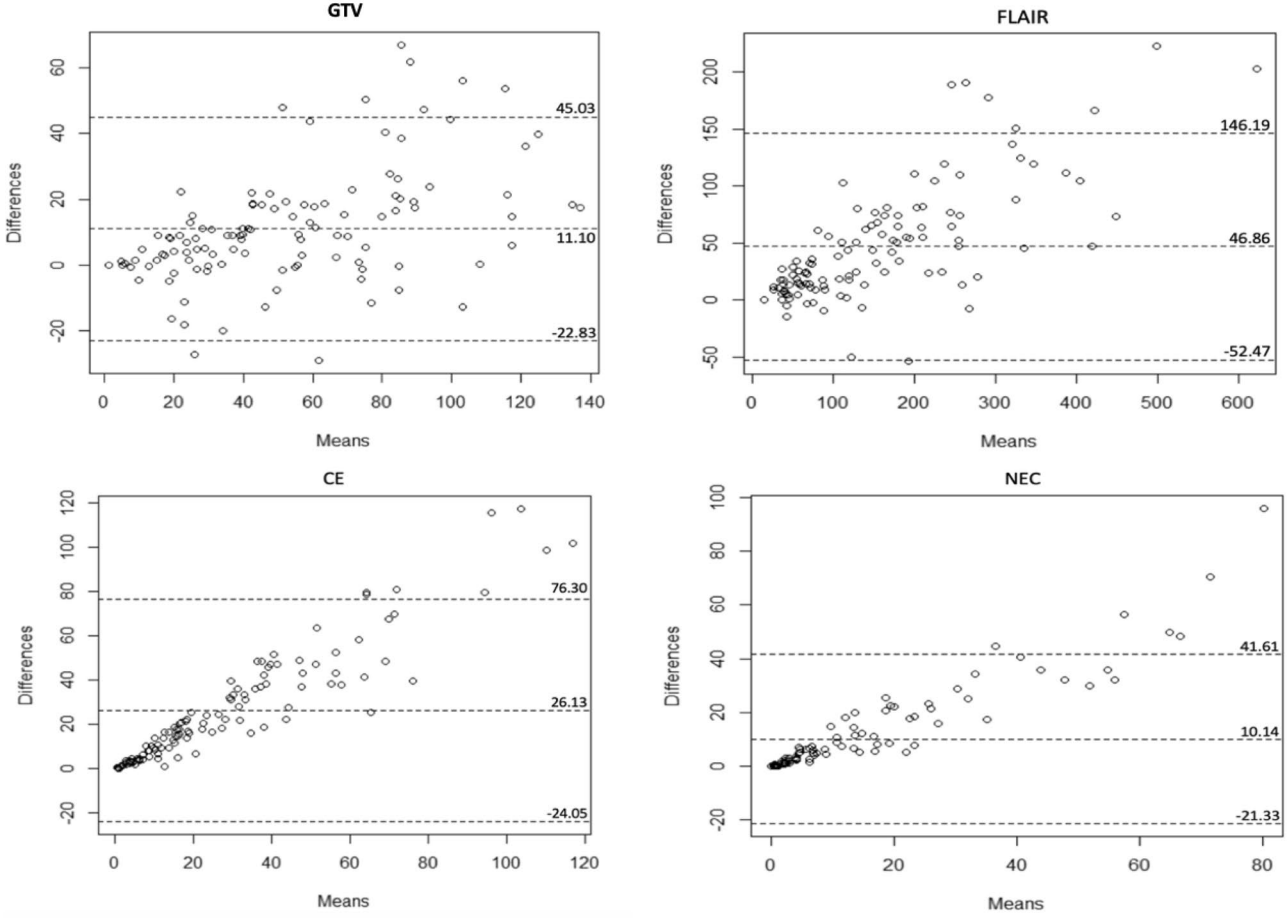
Bland Altman plots of the difference between the calculated volume and the measured volume in cm^3^. *CE* contrast-enhancement, *GTV* gross target volume, *NEC* necrosis area.

A high correlation was observed between the CV and MV for the CE (r = 0.91, 95% CI: 0.87–0.94, p < 0.001), the NEC (r = 0.94, 95% CI: 0.91–0.96, p < 0.001), the GTV (r = 0.90, 95% CI: 0.85–0.93, p < 0.001) and the FLAIR (r = 0.95, 95% CI: 0.93–0.97, p < 0.001) (Fig. 1).

For the CE, the NEC, the GTV, and the FLAIR, the intraclass correlation coefficient (ICC) was 0.33 (95% CI: 0.16–0.49), 0.56 (95% CI: 0.42–0.67), 0.82 (95% CI: 0.75–0.87) and 0.84 (95% CI: 0.78–0.89), respectively, with a good inter-rater reliability for the GTV and the FLAIR, a moderate inter-rater reliability for the NEC and a poor inter-rater reliability for the CE.

### Response assessment agreement according to the RANO criteria

For the CE size evolution in percent estimated with CV method, one, three, 23 and 30 patients were classified as complete response (CR), partial response (PR), stable disease (SD) or progressive disease (PD), respectively. For the CE size evolution in percent estimated with the MV method, one, zero, 19 and 37 patients were classified as CR, PR, SD and PD, respectively (Table 2). A total of 41 (72%) patients were classified in the same category with the CV method and the MV method (Table 2). The kappa coefficient value between the CV and MV was 0.66 (95% CI:0.45–0.87, p < 0.001), revealing substantial agreement between the two volume measurement methods to classify patients according to the response to treatment into four groups.

**Table 2:** Agreement in the RANO response assessment category between the two methods.

RANO response assessment category		CE Measured volume (manual delineation)				
		CR	PR	SD	PD	Total
CE calculated volume (ellipsoid formula)	CR	1	0	0	0	1
	PR	0	0	3	0	3
	SD	0	0	13	10	23
	PD	0	0	3	27	30
	Total	1	0	19	37	57

*CE* contrast-enhancement; *CR* complete response; *PD* progressive disease; *PR* partial response; *SD* stable disease.

### Survival analysis

**Figure 2:**
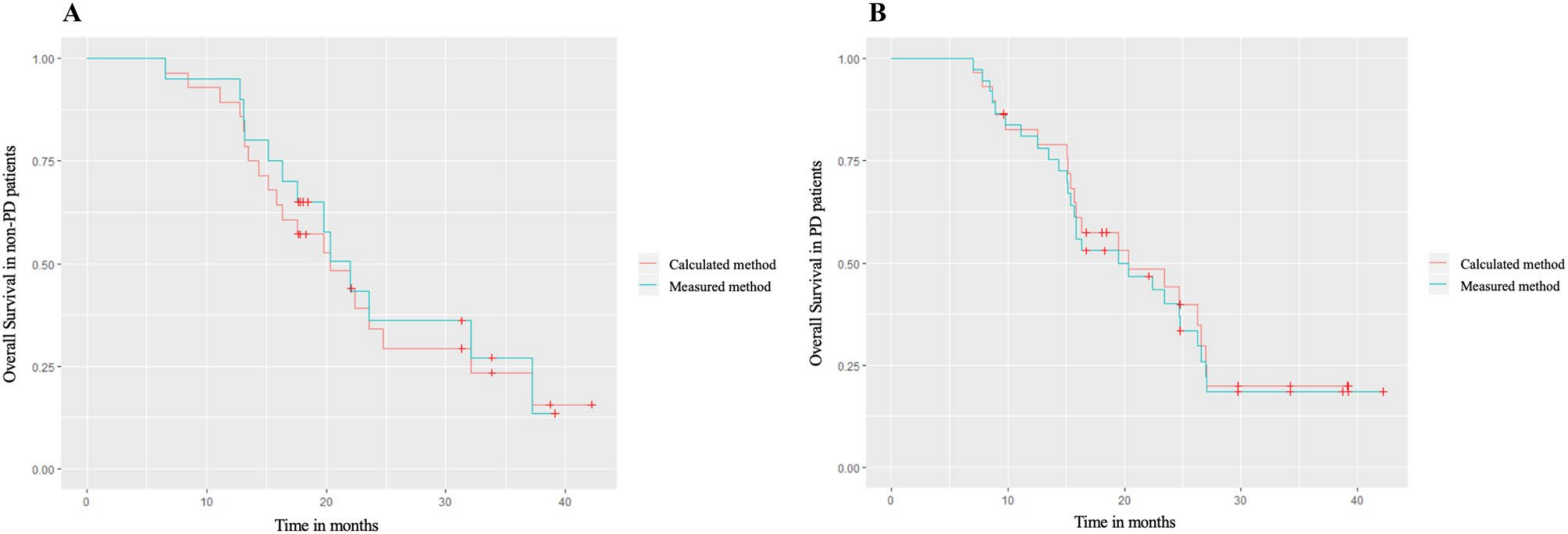
Overall survival according to the volume measurement methods. (**A**) Patients considered non-PD and (**B**) patients considered PD. *OS* overall survival, *PD* progressive disease.

With the CV method, 30 patients were classified as PD, and 27 patients were classified as non-PD; with the MV method, 37 patients were classified as PD, and 20 patients were classified as non-PD (p < 0.001). With the CV method, 20 patients died in the PD group, and 20 patients died in the non-PD group. With the MV method, 27 patients died in the PD group, and 13 patients died in the non-PD group. According to the CV or MV method, there was no significant difference in the median OS for PD patients (20.3 months and 19.5 months, respectively; HR = 1.096, 95%CI 0.615–1.954, p = 0.756), and for non-PD patients (20.4 months and 22.0 months, respectively; HR = 0.890, 95%CI: 0.442–1.790, p = 0.743) (Fig. 2).

## Discussion

This study compared the use of geometric model and manual segmentation to evaluate GBM volume and its evolution after specific treatment. The investigations revealed that the CV, with an ellipsoid formula based on tumor diameters, and the MV obtained by manual contouring, had a high correlation for all tumor compartments size estimation with a high agreement to classify patients in RANO treatment response group. Except for GTV and FLAIR that are continuous volumes on imaging, ellipsoid calculations of necrosis and CE were more than twice the MV measurements. The mean difference between calculated and measured volume for CE, NEC, GTV and FLAIR was 115.2%, 139.9%, 21.7% and 37.9%, respectively. This could be explained by the fragmented, shape size, blurred borders and irregular presentation of these volumes^14^ leading to sum several ellipsoid calculations, consequently, summing more higher volumes inherent to ellipsoid calculation. However, the difference of these ellipsoid volumes with MV measurements were lower than differences obtained with spheroid and rectangle calculations (data not shown). Consequently, a coefficient correlation from 0.90 to 0.95 was obtain for all the regions of interest but, the intermethod agreement was poor for CE (ICC = 0.33) and NEC (ICC = 0.56). The literature showed comparable results. Sreenivasan et al*.* conducted a retrospective study comparing manual segmentation, considered as reference, and four linear methods (sphere, cylinder, ellipsoid, simplified ellipsoid) in 44 patients (15 GBM). showed a high agreement with ellipsoid formula and manual segmentation (r = 0.81 or 0.86 according to the rater) for tumor volume measurement and inter-rater agreement for each method was high^14^. In the present study, one observer (a radiation oncologist) provided CV and MV measurements, in opposition to some studies where two or more observers delineation were used for comparison^14,16–18,24–26^. The current approach was original improving homogeneity and removed the inter-observer variability. Furthermore, all MRI scans were performed by one scan with the same imaging parameters that decrease inter-observer variability.

The 2D measurement was very simple and fast to use and was considered adapted in routine clinical practice without the need for specific software^11^. However, the intra- and inter-observer variabilities were high, and measurements could lack of objectivity and reproducibility^23,27^ and provide a worse estimation tumor response to treatment^24,25,28,29^. Moreover, the 2D measurement indirectly included the cystic and surgical cavity^30^, although the RANO criteria stated their exclusion^31^. With the recent development of novel therapies causing pseudoresponse and pseudoprogression, the treatment response needed more accuracy and reliability and less inter-observer variability^32^. For this reason, a volumetric approach sparked interest^33^. With a volumetric measurement, GBM boundaries were respected and cystic and necrotic areas were excluded^7^. To replace the 2D measurement method with simple and reproducible method, geometric models were studied and some authors showed the ellipsoid formula was more accurate than spheroid, cylinder or rectangular models^13,14,26^. For that, for the CV method, the ellipsoid formula was chosen as the geometric model to estimate the tumor volume for its simplicity in everyday clinical setting. However, some authors concluded ellipsoid formula remained insufficient with a higher intra- and inter-rater variability and less sensitivity to analyze early progression and small lesion evolution^13,27^.

For the MV method, manual segmentation was used. Although this method was time consuming^17,34,35^, expensive and potentially subjective, tumor segmentation was a complex task that needed much experience and competency for the appreciation of mixed areas, cystic and surgical cavities, necrosis, shape, and border enhancement that were not always well defined and reproducible from one software to another with semi-automated or fully automated methods^12,15,22^ but these software remain disputable^15,23^.

The GBM response to treatment was routinely evaluated by conventional MRI, with the change in tumor size. For a standardized response evaluation, the RANO criteria were used to classify patients^5^. Radiological criteria were based on contrast-enhancing lesion and FLAIR and excluded cystic and surgical cavities. However, some limitations persisted. The assessment of contrast-enhancing lesions was based on a 2D measurement, not on a volumetric approach. The FLAIR lesion assessment was not defined with a percentage of change and was neuroradiologist appreciation-dependent^36^. Ellingson et al. proposed modified RANO criteria to evaluate the radiological response with a volumetric approach, only considering contrast-enhancing lesions, with a threshold of 40% increase in volume for PD, without volume measurement recommendation^9^. In the current study, the classification of patients was exclusively based on CE volume changes, as suggested by modified RANO criteria.

Some authors studied the impact of volume measurement methods on the response to treatment assessment in GBM patients^11,16,17,22^. However, comparison were only between the different studied methods^17,22^, RECIST evaluation^16^, RANO response^11^. In this study, when modified RANO response groups were used according to the volume percentage changes, the CV and MV had a substantial agreement of 72% (K = 0.66) that revealed manual segmentation did not improve patient response determination according to modified RANO criteria versus ellipsoid model. This agreement was excellent for the two patients with CR and PR. Difference can be observed in SD and PR agreement where ellipsoid calculation was more “optimistic” than the MV evaluation. This could be the consequence of (i) the difference of initial values obtained by the two methods (i.e. ellipsoid and MV methods), (ii) progression was evaluated only on CE volumes and not in all regions of interest.

This current study revealed that there was no difference in OS for patients with PD or not according to the volume methods used. This could be explained by the fact that patients always relapse and the time between relapse and death is always short. Time of relapse has only a low impact on OS. Secondly, the number of patients in this series can also be a cause of the absence of difference. Thirdly, this could be a reason to use the ellipsoid method, because of this absence of change. However, in the series showing a significant difference between measurements and OS, number of patients were lower or equivalent to the number of our series, but the cut-off to conclude of a progression was highly different, leading comparison between series very challenging^11,37–40^.

As numerous study limitations in the literature, this study was a retrospective study with a relatively small sample size of population with a lack of statistical power. Another issue of the type of study was the lack of in vivo standard reference of brain tumor volume measurement and truth volume size unknowledge. Despite the development of numerous automated segmentation methods and more than 20 years of research, computer assisted methods remained challenging and the need of clinical research persist to homogeneous practice^41^. Finding a pertinent tool with high relevance in routine, easy to use and adapted to the therapeutic management and clinical trial design was a real challenge. In addition to the conventional MRI analysis, tumor size determination using advanced and multimodal MRI appeared promising^20,42,43^. To further improve the assessment of GBM, machine learning models were developed^44^. In fact, an MRI containing over a million voxels that constituted a complex “big data” management and deep learning methods for segmentation, survival prediction or brain tumor gradation was to develop^45,46^. Therefore, quantitative features as textural and geometric data could be explored, combined with genomics, proteomics and clinical data and compiled into diagnostic, prognostic, and therapeutic models^47^.

## Methods

This study was approved by the center’s institutional review board. All methods were performed in accordance with the relevant guidelines. Informed consent was obtained from all the patients included in this study.

### Population

A total of 139 patients with newly diagnosed and histologically confirmed GBM were identified between January 2015 and December 2017 and reviewed in this single-center retrospective study. Inclusion criteria consisted of (1) age 18 years or older, (2) histopathological confirmation of GBM, (3) completion of entire course of CRT with TMZ after maximal surgery according to the EORTC/NCIC protocol^5^, and (4) MRI follow-up until progression. Thirty-eight patients were excluded because of hypofractionated RT schedules, 15 for another chemotherapy protocol (bevacizumab), eight died before progression, seven had no progression at the time of data collection, five were lost to follow-up, five had gliosarcoma histological conclusion, three had no MRI examination during the follow-up, and one had a history of cerebral irradiation. Finally, 57 patients were included in the study. Patient ages ranged from 24 to 81 years with a median age of 62 years. Forty patients (70%) were male and 17 patients (30%) were female. Two MRIs per patients were evaluated corresponding to the MRI performed before CRT (dosimetric MRI performed 4–6 weeks after surgery to plan radiotherapy) and the MRI where a suspicion of a first progression was diagnosed. The examination was performed on a Signa Excite HDx 3.T™ system (GE Healthcare, Milwaukee, WI) with an 8-channel dedicated head coil. The MRI scanning protocol included pre- and postcontrast 1-mm, 3-dimensional (3D) volumetric T1-weighted multi-echo magnetization-prepared rapid-acquisition gradient echo (MPRAGE) sequences, and a T2-FLAIR images. MRI showing a suspicion of progression was performed at a mean of 23.6 weeks after the completion of CRT.

### Recorded data

**Figure 3:**
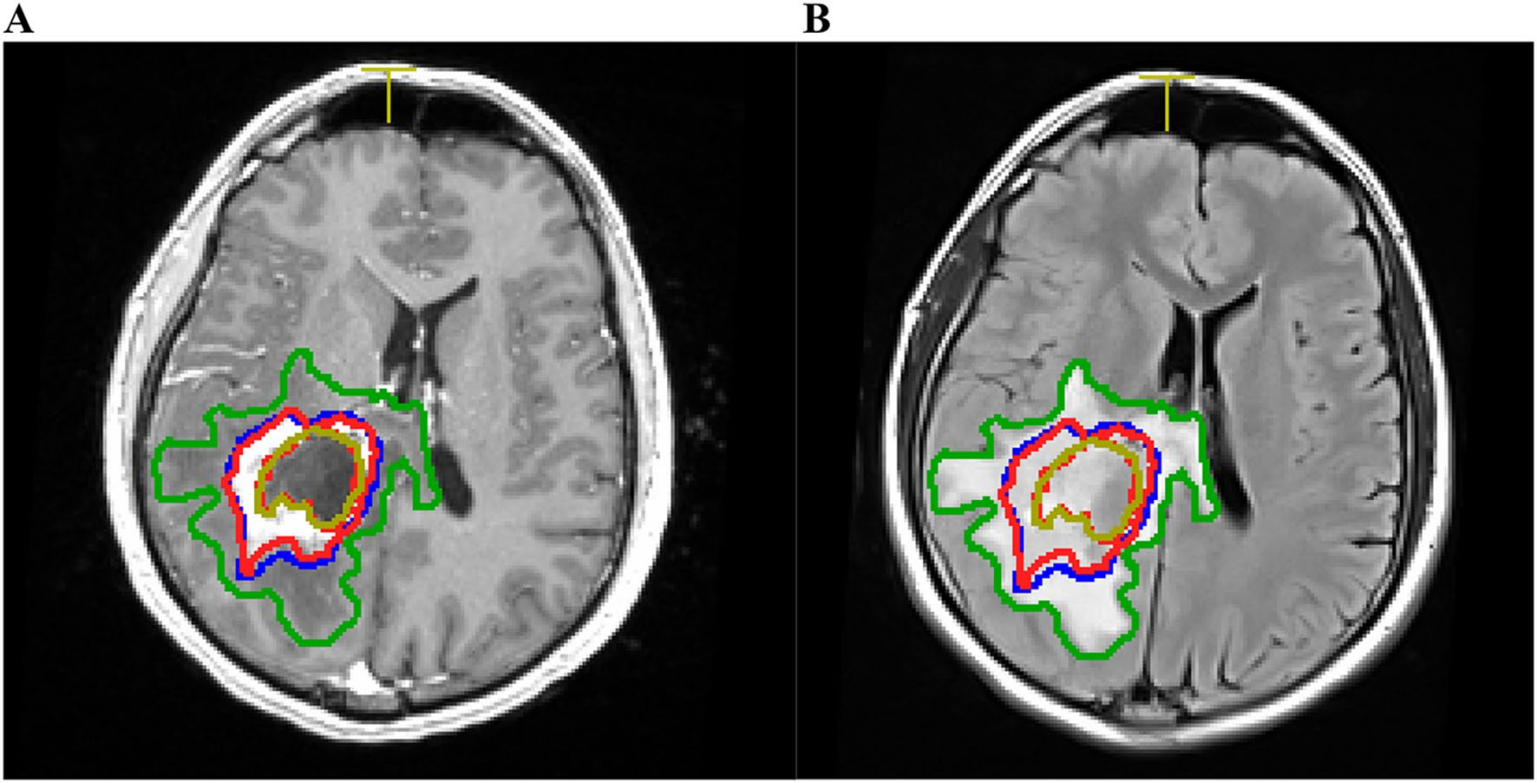
Manual segmentation of the glioblastoma compartments on T1-weighted contrast-enhanced MRI and T2-Flair MRI sequences. GTV (gross target volume) in blue, CE (contrast-enhanced region) in red, NEC (necrosis areas) in yellow, FLAIR in green in axial slice of (**A**) T1-weighetd contrast-enhanced MRI sequence, (**B**) T2-Flair MRI sequence.

For each MRI, the volume of different GBM regions of interest was evaluated (Fig. 3). On the T1-weighted contrast-enhanced MPRAGE sequence, the CE, the NEC and the GTV which included the CE, the NEC and the surgical cavity, were obtained. On the T2-FLAIR sequence, the volume of the edema area (FLAIR) was measured. On the MRI performed before CRT, one, two or three CE areas were seen in 48, five and four patients, respectively. When region of interest was fragmented, we performed an ellipsoid calculation for each fragment and the overall ellipsoid calculation was the sum of all the ellipsoid fragments calculations.

Two methods of volume measurements were used to define the volume of different GBM regions of interest and compared.

The CV for each compartment of the tumor was obtained with the ellipsoid volume formula: π/6*D1*D2*D3, where D1, D2 and D3 corresponded to the largest diameter of the compartment measured in three-dimensional plans (axial, sagittal and coronal reformations).

The MV resulted from the manual delineation in all MRI slices, (slice per slice) of each compartment computed with FocalSim™ (Elekta^®^, Stockholm, Sweden) contouring software. After contouring, the volume was automatically calculated by the software for each compartment.

For patients with multifocal lesions, all lesions were measured with the two methods separately and summed for the comparison. For the CE, the analyzed volume corresponded to the sum of the measurable lesions (which had at least two diameters greater than 10 mm) according to the RANO criteria.

The measurements were performed in by only one radiation oncologist resident with 6 years of experience (CL) and corrected by two reviewers with over 20-years of experience, a neuroradiation oncologist expert (GN) and a neuroradiologist expert (JMC). Any disagreements between the two reviewers were resolved through discussion between the three protagonists and potential corrections were consensually adopted. Finally, all measurements were approved by the two reviewers.

### Evaluation of the response according to the RANO criteria

Only for the response to treatment classification was the MRI showing the best response to treatment examined in seven patients (data not shown).

Assessment response category, CR, PR, SD or PD, defined according to the RANO^5^ and the modified RANO criteria^10^, were applied only on the CE comparing the MRI performed before CRT and the MRI showing a suspicion of progression for 50 patients and the MRI showing the best response after treatment and the MRI showing a suspicion of progression for seven patients^3,5,6,9,17,28^. For each patient, the classification based on the CV and the classification based on the MV were compared. CR, PR, SD and PD were defined according to Ellingson et al*.* (CR 100% decrease; PR ≥ 65% decrease; PD ≥ 40% increase; SD 40–65%)^9^.

### Statistical analysis

The comparison was performed in volume (cm^3^) and percent variations between the two methods of measurement. The correlation between the two volume measurement methods for each compartment was analyzed with Pearson correlation coefficients and 95% confidence intervals (95%CIs). For each type of compartment, the ICC estimated the interrater reliability of measurements as follows: poor reliability for ICC < 0.50, moderate reliability for ICC 0.50–0.75, good reliability for ICC 0.75–0.90 and excellent reliability when the ICC > 0.90. The comparison of the two measurement methods was represented by a Bland–Altman plot for each compartment. The agreement of the patient’s response to treatment category of the two volume measurement methods was assessed using Cohen’s weighted kappa statistic with the 95% CI with the kappa value ranging from −1 to + 1. A kappa value between 0.01 and 0.20 indicated no or slight agreement, between 0.21 and 0.40 indicated fair agreement, between 0.41 and 0.60 indicated moderate agreement, between 0.61 and 0.80 indicated substantial agreement and between 0.81 and 1.00 indicated almost perfect agreement.

A survival analysis was performed to evaluate the impact of the volume measurement methods on OS according to the response to treatment expressed by the radiological RANO classification (CE volume evolution). Patients were classified as PD or non-PD (CR, PR or SD). OS was determined from the date of pathological diagnosis to death or the last follow-up. OS in PD and non-PD patients was estimated by a log-rank test, and Kaplan–Meier survival curves^48,49^ were drawn for each group according to the measurement methods. A Cox regression analysis^50^ was performed to compare the two measurement methods to predict OS, with the determination of hazard ratios (HRs) and their 95% confidence intervals (95% CIs).

Statistical calculations were performed with R version 3.6.1 software (https://www.r-project.org).

### Ethics approval

This study received institutional ethics board approval from the research committee of the ICANS comprehensive cancer center.

### Consent for participate/consent for publication

Written informed consent was obtained from the patient for the publication of this report.

## Conclusions

The GBM evaluation should be not ambiguous or complex for clinical management and clinical trials, and a volume approach seemed more realistic. The development, standardization and accessibility of segmentation methods should be encouraged. The current study showed a high concordance between manual segmentation and the ellipsoid formula to define the volumes of GBM compartments with a good agreement to classify the patient response to treatment according to the four RANO groups, suggesting the use of the ellipsoid formula in clinical practice. However, CV measurements were significantly larger than MV measurements and the inter-rater variability for CE volume definition was poor. For that the treatment response categorization of patients should be performed with caution using the ellipsoid formula and segmentation methods must be preferred to make therapeutic decision.

## Data Availability

The datasets generated during and/ or analyzed during the current study are available from the corresponding author on reasonable request.

## Abbreviations

CE: Contrast enhancement
CR: Complete response
CRT: Chemoradiotherapy
CV: Calculated volume
GBM: Glioblastoma
GTV: Gross target volume
HR: Hazard ratio
ICC: Intraclass correlation coefficient
MRI: Magnetic resonance imaging
MV: Measured volume
NEC: Necrosis area
OS: Overall survival
PD: Progressive disease
PFS: Progression free survival
PR: Partial response
RANO: Response assessment in neuro-oncology
SD: Stable disease
